# Preparation and In Vitro Simulated Digestion Study of Coembedded Microcapsules Containing Pomegranate Active Extract and Probiotics

**DOI:** 10.1155/ijfo/1979133

**Published:** 2026-09-13

**Authors:** Yanzi Ge, Jiayi Hu, Shu Liu, Guang Yang, Qingyun Hua, Qiran Gu, Tianyi Mao, Xiaoxu Xue, Bo Yu, Yaowei Fang

**Affiliations:** ^1^ School of Marine Food and Biological Engineering, Jiangsu Ocean University, Lianyungang, China, hhit.edu.cn; ^2^ Kangda College, Nanjing Medical University, Nanjing, China, njmu.edu.cn; ^3^ Sino-German Joint Research Institute, Nanchang University, Nanchang, China, ncu.edu.cn

**Keywords:** pomegranate extract, probiotics, sodium alginate

## Abstract

The efficient delivery of viable probiotics to the intestine remains a major challenge due to their sensitivity to gastrointestinal conditions, whereas pomegranate‐processing by‐products represent an underutilized source of bioactive polyphenols. In this study, a dual‐functional codelivery system of pomegranate extract and probiotics was constructed to enhance the survival rate of *Limosilactobacillus fermentum* FUA033 in the gastrointestinal environment. Pomegranate peel and seed active substances were extracted using an ultrasound‐assisted deep eutectic solvent. Microcapsules were prepared by the ion‐gel method, and the encapsulation efficiency reached 90.23% under the optimal process. The microcapsules exhibited a monodispersed spherical morphology of 3.25 ± 1.06 mm diameter and characteristic FTIR peaks (1633–1641 cm^−1^), confirming structural integrity. The results of the XRD analysis revealed a crystalline phase (2*θ* = 31.52°). PE‐FUA033 microcapsules significantly improved probiotic viability during storage (with increases of 13.51% and 23.41% at 4°C and 25°C, respectively) and simulated gastrointestinal digestion (32.41% greater survival than the PE‐free controls), providing a new strategy for the development of probiotic functional foods.

## 1. Introduction

Probiotics are live microorganisms that, when administered in adequate amounts, confer health benefits on the host by colonizing the gut and modulating the microbiota structure [1]. They primarily include genera such as *Lactobacillus*, *Bifidobacterium*, *Saccharomyces*, and *Streptococcus*, and their applications have expanded from traditional fermented dairy products to functional foods and disease prevention [2]. To ensure effective intestinal colonization, the initial viable cell density in formulations needs to be > 10^9^ CFU/g. Additionally, the viable probiotic count reaching the human intestine must be at least > 10^6^–10^7^ CFU/g [3]. However, probiotics are susceptible to external environmental stress, such as high temperature, low pH, oxygen, gastric acid, bile salts, and digestive enzymes, during intestinal colonization [4]. Therefore, ensuring probiotic quantity and viability poses a significant challenge.

Microencapsulation technology significantly increases probiotic resistance to harsh environments such as gastric acid and bile salts by constructing protective barriers using functional wall materials [5]. This technology can be classified into physical, chemical, and physicochemical methods based on its preparation principles [6]. Physical methods include spray drying and extrusion [7], chemical methods primarily involve emulsification [8], and physicochemical methods encompass complex coacervation and ionic gelation [9]. Common wall materials include polysaccharides and proteins; polysaccharides such as sodium alginate, chitosan, and starch [10]; and proteins such as casein and whey protein [11]. SA hydrogels form a pH‐responsive three‐dimensional network structure through cross‐linking reactions between –COOH groups and Ca^2+^ ions from CaCl_2_ [12], effectively providing gastric acid protection and colon‐targeted release. Polyphenols can effectively improve probiotic viability during drying, storage, and digestion, whereas probiotics can increase the stability and bioavailability of polyphenols [13]. Pomegranate peel and seeds, as typical food processing byproducts, are rich in dietary fiber, punicalagin, ellagic acid, and flavonoids [14]. They have antimicrobial, antioxidant, and antitumor properties and can improve the physical properties of microcapsules [15]. The encapsulation of polyphenols in sodium alginate cryogels can also increase their release rate, mechanical strength, and stability [16]. Therefore, the use of pomegranate peel and seed extracts, which are rich in polyphenols and have synergistic effects with probiotics, as raw materials for probiotic microencapsulation, is an effective strategy for enhancing the overall performance of encapsulation systems.

Our research group previously screened the probiotic strain *Limosilactobacillus fermentum* FUA033, which efficiently converts ellagic acid into urolithin A. UA has multiple biological effects, including antimicrobial, anti‐inflammatory, antitumor, antioxidant, and antiaging effects, and can cure intestinal diseases [17]. Fermenting juices from strawberries, raspberries, blackberries, and mulberries with this strain successfully converted EA in the berries into UA, enhancing the bioactivity, improving the flavor of the juices [18], and demonstrating its potential for developing novel probiotic products. Additionally, strain FUA033 is safe, acid and bile salt–tolerant, and adheres to the intestinal epithelium. Its survival rates were found to be 62*%* ± 1.83*%* after 6 h in simulated gastric acid and 65*%* ± 2.09*%* after 6 h under bile salt stress. The surface hydrophobicity and autoaggregation of the strain were positively correlated with its in vivo colonization ability [19]. Compared with the reported hydrophobic rate and self‐coagulation rate of commercial *Bifidobacterium longifolium* BB536 at 16.8*%* ± 0.8*%* and 6.8*%* ± 0.7*%* [20], the hydrophobic rate and self‐coagulation rate of FUA033 were 33.2*%* ± 1.5*%* and 10.4*%* ± 0.5*%*, respectively, indicating that it can strongly promote initial mucosal adhesion and accelerate biofilm formation.

To further enhance the viability and metabolic activity of the FUA033 strain during gastrointestinal delivery, this study is aimed at developing a bifunctional codelivery system by integrating an efficient extraction method with a sodium alginate–calcium chloride cross‐linking matrix. Specifically, we employed an ultrasound‐assisted natural deep eutectic solvent to extract polyphenol‐rich components from pomegranate peel and seeds. The extract was then coencapsulated with FUA033 into alginate microcapsules via ionic gelation. The preparation parameters (alginate concentration, CaCl_2_ concentration, extract concentration, and curing time) were optimized to maximize encapsulation efficiency. The resulting microcapsules were characterized by morphology, FTIR, XRD, and their performance in terms of storage stability and simulated gastrointestinal digestion was evaluated. This approach is expected to achieve gastric protection and colon‐targeted release of both probiotics and polyphenols, synergistically enhancing their health benefits while promoting the valorization of pomegranate processing by‐products.

## 2. Materials and Methods

### 2.1. Materials and Reagents

*Lactobacillus fermentum* FUA033, which was isolated by our laboratory, was stored at the China General Microbiological Culture Collection Center (Accession Number: CGMCC No.28447). Pomegranates (Jiawang District, Xuzhou City, China), Wilkins‐Chalgren Anaerobe WAM (Shanghai Jizhi Biochemical Technology Co. Ltd), sodium alginate, CaCl_2_, NaCl, NaHCO_3_, KH_2_PO_4_ and sodium citrate are all of analytical grade (Shanghai Aladdin Biochemical Technology Co. Ltd). Pepsin (Cas No: 9001‐75‐6, Sinopharm Group Chemical Reagent Co. Ltd), trypsin (Cas No: 9002‐07‐7, Sinopharm Group Chemical Reagent Co. Ltd, China), and ox bile salt (National Medicine Code: 69005262, Sinopharm Group Chemical Reagent Co. Ltd, China).

### 2.2. Instruments

The instruments used were as follows: UV‐1000 Ultraviolet Spectrophotometer (Shanghai Aoyi Instrument Co. Ltd), Multifuge X 3R Benchtop High‐Speed Refrigerated Centrifuge (Thermo Fisher Scientific, United States), Regulus‐8100 Cryo Field Emission Scanning Electron Microscope (Hitachi, Ltd), Nicolet iS10 Fourier Transform Infrared Spectrometer (Thermo Fisher Scientific), X′Pert POWDER Powder Diffractometer (PANalytical B.V., the Netherlands), SW‐CJ‐1FD Clean Bench (Suzhou Huayu Purification Equipment Co. Ltd), MLDZX‐50L Vertical High‐Pressure Steam Sterilizer (Shanghai Shen′an Medical Instrument Factory), and GRX‐9203A Microbial Constant‐Temperature Incubator (Shanghai Fuma Experimental Equipment Co. Ltd).

### 2.3. PE Extraction and Determination of TPC

#### 2.3.1. Preparation of DES

Urea and betaine were weighed at a 1:2 molar ratio and placed in a flask with ultrapure water; the water content was adjusted to 40% (w/w). The mixture was magnetically stirred at 80°C for 6 h until a homogeneous transparent liquid formed [21]. The eutectic mixture was cooled to 25°C for later use.

#### 2.3.2. PE Extraction

Whole pomegranate seeds were wrapped in gauze and scrubbed with deionized water at 25^°^C ± 2^°^C to thoroughly remove surface pulp and mucus. Pomegranate peels and cleaned seeds were dried in a fume hood at 55°C until a constant weight was reached. After grinding with a high‐speed grinder, the powder was sieved through a 40‐mesh sieve to remove large particles. The mixed powder was collected and stored at −20°C. Next, 1.0 g of pomegranate powder was mixed with 20 mL of DES and sonicated at 25°C for 15 min. The reaction was immediately stopped in an ice bath. After centrifugation at 5000 × g for 10 min, the supernatant was collected as PE [22].

#### 2.3.3. Determination of the TPC in PE

A total of 1.0 g of pomegranate powder was mixed with 30 mL of the prepared DES and stirred thoroughly at a solvent‐to‐solid ratio of 30:1 mL/g [23]. Extraction was performed at 60°C for 30 min under ultrasonication. After centrifugation at 8000 r/min for 15 min, the supernatant was collected.

A 0.04‐mg/mL gallic acid standard solution was prepared. Serial dilutions yielded solutions with concentrations of 0.005, 0.010, 0.015, 0.020, and 0.025 mg/mL. A total of 1.0 mL of each solution was transferred to a 10‐mL stoppered tube and mixed with 2 mL of Folin–Ciocalteu reagent, followed by 2 mL of 10% Na_2_CO_3_ solution. After thorough mixing, the mixture was kept in the dark at room temperature for 60 min, and the absorbance was measured at 760 nm.

**Figure 1:**
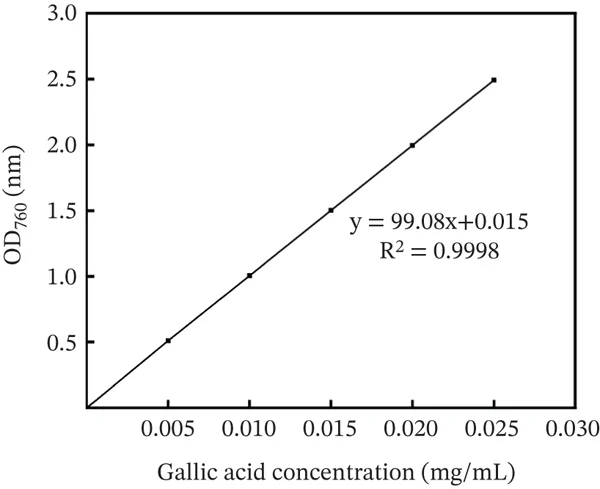
Gallic acid standard curve.

Linear fitting was performed with the mass concentration (X) of gallic acid as the abscissa and the absorbance value (Y) as the ordinate, and the standard curve regression equation was obtained as y = 99.08x + 0.015 (Figure 1).

The pomegranate extract was diluted appropriately, and its absorbance was measured following the same procedure. The polyphenol concentration was calculated using a linear equation, and the TPC in the powder was expressed as mg gallic acid equivalents (GAE)/g.

TPCmgGAE/g=x×v×nm

Here, x indicates the polyphenol concentration in the sample (mg/mL), v indicates the total volume of the solution (mL), n indicates the dilution factor, and m indicates the mass of pomegranate peel powder (g).

### 2.4. Preparation and Optimization of PE‐FUA033 Microcapsules

#### 2.4.1. Preparation of FUA033 Cell Suspension

Strain FUA033 stored at −80°C was activated by streaking on MRS agar plates, inoculated into MRS liquid medium, and cultured anaerobically at 37°C for 24 h. Cells were collected by centrifugation at 5000 × g for 10 min (4°C), washed twice with 0.9% sterile saline, and resuspended in 0.9% sterile saline to a final concentration of 1 × 10^10^ CFU/mL [24].

#### 2.4.2. Preparation of SA Solution

SA powder was added to deionized water at the target concentration. The mixture was heated at 80°C with constant magnetic stirring at 600 rpm for 90 min to obtain a homogeneous transparent SA solution, which was cooled to 25°C for later use [25].

#### 2.4.3. Preparation of PE‐FUA033 Microcapsules

Initially, PE, FUA033 cell suspensions, and SA solutions were thoroughly mixed. Using a 22‐G sterile syringe, the mixture was dripped steadily from a height of 10 cm above the liquid surface into a CaCl_2_ solution. The droplets were kept undisturbed for complete cross‐linking and curing [26]. The microcapsules were collected, washed three times with sterile water to remove free Ca^2+^ and unencapsulated cells, and finally freeze‐dried into a powder [27]. A control group without PE was prepared following the same steps.

The optimal preparation conditions for PE‐FUA033 microcapsules were determined by varying the SA concentration (0.6%, 0.8%, 1.0%, 1.2%, and 1.4% (w/v)), CaCl_2_ concentration (0.6%, 0.8%, 1.0%, 1.2%, and 1.4% [w/v]), PE concentration (0.5%, 1.0%, 1.5%, 2.0%, and 2.5% [v/v, relative to SA solution]), and curing time (20, 30, 40, 50, and 60 min).

#### 2.4.4. Determination of Microcapsule Encapsulation Efficiency

A 3% (w/v) sodium citrate solution was used as the decapsulation solution [28]. PE‐FUA033 microcapsules (1.0 g) were added to 9 mL of decapsulation solution and shaken at 37°C and 100 rpm for 30 min for lysis. A total of 0.1 mL of lysis buffer was used for gradient dilution, and a 10‐fold series dilution was performed with sterile normal saline. Appropriate dilutions were spread onto MRS agar plates and incubated anaerobically at 37°C for 48 h for viable cell counting [29].

EE%=NN0×100%

Here, EE indicates the encapsulation efficiency (%), N indicates the number of viable cells in 1 g of microcapsules (CFU/g), and N_0_ indicates the total number of viable cells before encapsulation (CFU/mL).

### 2.5. Characterization of Microcapsules

#### 2.5.1. Morphological Observation and SEM

The prepared microcapsules were spread on a glass plate. The diameter of 30 randomly selected microcapsules was measured using a Vernier caliper, and the average diameter was calculated. Dehydrated freeze‐dried microcapsules were analyzed using a QUANTA 250 FEG scanning electron microscope [30]. The samples were mounted on stubs, sputter‐coated with gold, and observed at an accelerating voltage of 20 kV. SEM images were captured and saved at magnifications of 50×, 2500×, and 5000×.

#### 2.5.2. Fourier Transform Infrared Spectroscopy Analysis

In a dry environment, a small amount of dried sample was mixed with KBr at a mass ratio of 1:200 and ground thoroughly in an agate mortar. The mixed powder was placed in a tablet press and maintained at a pressure of 8 tons for 2 min to prepare semitransparent thin sheets with a diameter of 13 mm and a thickness of 0.5 mm [31]. FTIR spectra were obtained using a Nicolet‐iS10 spectrometer with a resolution of 4 cm^−1^, 32 scans, and a wavenumber range of 400–4000 cm^−1^. The atmospheric background was subtracted before each sample was scanned [32].

#### 2.5.3. X‐Ray Diffraction Analysis

The crystal structure and composition of the freeze‐dried microcapsules were analyzed via X‐ray diffraction. The samples were ground to a particle size of < 50 *μ*m and packed into sample holders via the pressing method, and the surface was leveled. The samples were continuously scanned using Cu Ka radiation, with a 2*θ* diffraction angle ranging from 5° to 50° and a scanning speed of 2°/min [33].

### 2.6. Storage Stability of Microcapsules

The microcapsules were stored at 4^°^C ± 1^°^C and 25^°^C ± 1^°^C. Samples were collected every 7 days. Then, 1 g of microcapsules was mixed with 9 mL of decapsulation solution, vortexed until complete disintegration and cell release, serially diluted, and subjected to viable cell counting to evaluate stability [29]. Free unencapsulated cells served as the control.

### 2.7. In Vitro Simulated Gastrointestinal Digestion

To prepare simulated gastric fluid, 0.35 g of pepsin and 0.20 g of NaCl were dissolved in 100 mL of sterile water. The pH was adjusted to 2.0 ± 0.1 using sterile HCl solution, and the solution was sterilized by passing it through a 0.22‐*μ*m microporous filter [34]. To prepare simulated intestinal fluid, 0.68 g NaHCO_3_, KH_2_PO_4_, and 0.04 g NaCl were dissolved in 100 mL of sterile water. Next, 1 mg of trypsin and 0.3 g of ox bile salt were added, and the mixture was stirred. The pH was adjusted to 6.8 ± 0.1 using sterile NaOH solution, and the solution was sterilized by passing it through a 0.22‐*μ*m filter [35].

The microcapsules were mixed with SGF at a 1:9 (w/v) ratio and incubated in a 37°C shaker at 120 rpm for 2 h. The microcapsules were removed and rinsed three times with 0.9% sterile saline to remove surface gastric fluid. One part was placed in 3% (w/v) decapsulation solution for wall dissolution. The other part was transferred to SIF at a 1:9 (w/v) ratio and incubated at 37°C and 100 rpm for 3 h, followed by viable cell counting to analyze the protective effect at different stages [36]. Free unencapsulated FUA033 was used as a control and subjected to the same simulated digestion procedure.

### 2.8. Data Analysis

All experiments were performed in triplicate. The data were analyzed using SPSS 22.0 and Design‐Expert software. Graphs were plotted using Origin 2022 software. All results were considered to be statistically significant at *p* < 0.05.

## 3. Results and Discussion

### 3.1. Determination of the TPC in PE

Under the optimized extraction conditions, the total phenol content obtained from pomegranate peel and seeds was 30.68 ± 0.12 mg GAE/g. In contrast, the total phenol amounts obtained by the traditional reflux extraction method and the impregnation method at the same extraction time were 21.32 ± 0.05 and 20.44 ± 0.05 mg GAE/g, respectively [37]. These findings indicate that the natural eutectic solvent combined with the ultrasonic‐assisted extraction method is significantly better than the traditional water–ethanol extraction method in terms of extraction efficiency. Additionally, the NaDES‐UAE method also has the advantage of a relatively low extraction temperature. This not only helps maintain the structural stability of heat‐sensitive active components, reducing the risk of degradation, hydrolysis, or oxidation, but also contributes to lowering the energy consumption during the extraction process. It is particularly suitable for the extraction of temperature‐sensitive bioactive components.

### 3.2. Optimization Results for PE‐FUA033 Microcapsule Preparation

**Figure 2:**
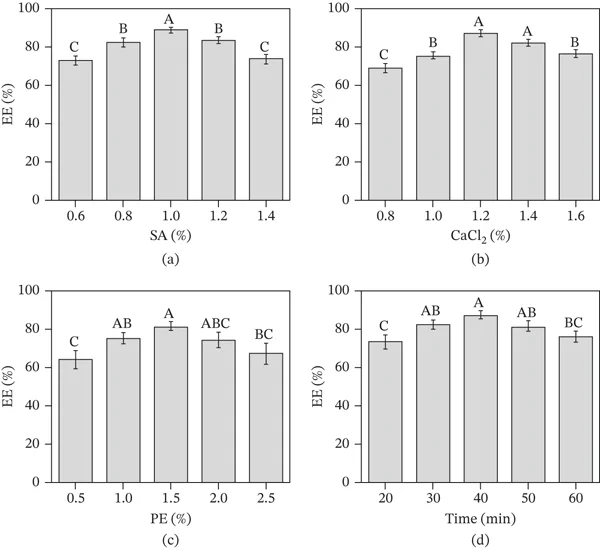
Effects of different factors on the encapsulation efficiency of PE‐FUA033 microcapsules: (a) sodium alginate concentration, (b) calcium chloride concentration, (c) PE concentration, and (d) curing time. Different lowercase letters (a–d) above the bars indicate statistically significant differences (*p* < 0.05).

The results of single‐factor optimization indicated that the concentration of SA significantly affected the encapsulation efficiency of the microcapsules (Figure 2a). When the SA concentration increased from 0.6% to 1.0% (w/v), the EE reached its peak value 88.67*%* ± 1.24*%*. If the SA concentration is too low, the gel network structure will be weak. During the filtration process, the microcapsules are prone to disintegration, resulting in a bacterial loss rate exceeding 25%. However, if the SA concentration is too high, it will cause the “tailing” phenomenon due to the overly fast exchange rate between Na^+^ and Ca^2+^, hindering the uniform molding of microcapsules [10]. The concentration of calcium chloride also significantly affects the packaging efficiency (Figure 2b). When its concentration was 1.2%, the EE was the highest 83.36*%* ± 1.55*%*. When the concentration is too low, an insufficient supply of Ca^2+^ leads to inadequate ion exchange, a loose cross‐linked network structure, and a poor embedding effect. When the concentration is too high, the gel will cure too quickly, resulting in cracks on the surface of the microcapsules, thereby reducing structural integrity and encapsulation efficiency [38]. The amount of PE added also strongly affects the EE (Figure 2c). When the PE concentration was 1.5%, the EE reached the optimal level 80.80*%* ± 2.12*%*. An appropriate amount of PE can prolong the survival of probiotics in the gastric acid environment through its polyphenol components and exert a protective effect [39]; however, excessive PE competes with the cells for binding sites in the SA‐Ca^2+^ cross‐linked network, interfering with the formation of microcapsules and hindering the process. The curing time is also a key parameter affecting the packaging effect (Figure 2d). At a curing time of 40 min, the EE reached 86.42*%* ± 1.62*%*. A very short duration may lead to insufficient cross‐linking, causing leakage of the bacteria. If the time is too long, the gel network becomes quite dense. Although it can protect the stomach, it may hinder targeted release in the colon [7].

The optimal preparation conditions for the PE‐FUA033 microcapsules were determined through single‐factor experiments as follows: SA solution concentration, 1.0% (w/v); calcium chloride solution concentration, 1.2% (w/v); PE concentration, 1.5% (w/v); curing time, 40 min. After comprehensive optimization, the EE increased to 90.23*%* ± 1.42*%*. The gradual optimization of the above parameters confirmed that the SA cross‐linking strength, ion exchange kinetics, compatibility of PE active ingredients, and curing time are key factors affecting the EE.

### 3.3. Structural Characterization of PE‐FUA033 Microcapsules

#### 3.3.1. Morphology and SEM

**Figure 3:**
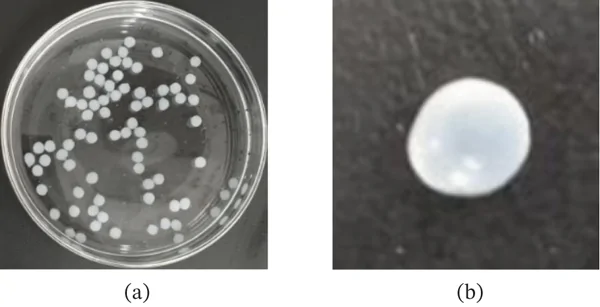
Appearance of microcapsules. (a) and (b), respectively, represent multiple microcapsules and a single microcapsule.

**Figure 4:**
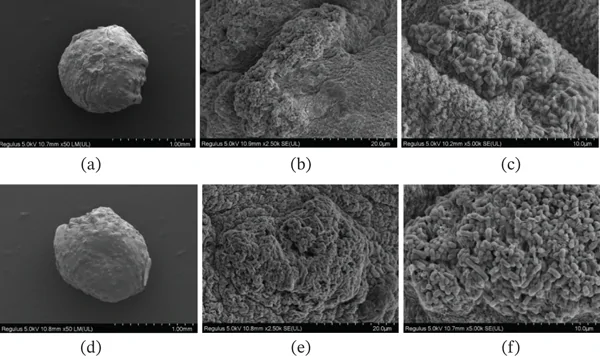
SEM images of microcapsules. (a–c) FUA033 microcapsules without PE at 50×, 2500×, and 5000×, SEM images of microcapsules. (a–c) FUA033 microcapsules without PE at 50×, 2500×, and 5000× magnification, respectively.

The microcapsules exhibited a spherical morphology with uniform particle size distribution (Figure 3), and the measured average diameter was 3.25 ± 1.06 mm. The spherical shape and uniform size are critical for consistent flow properties, reproducible release behavior, and predictable gastrointestinal transit kinetics. As shown in Figure 4, the SEM images revealed distinct structural features between the two formulations. The PE‐FUA033 microcapsules displayed a lamellar adsorption layer on the surface (Figure 4d,f), formed via electrostatic adsorption and hydrophobic association: negatively charged polyphenolic groups in PE formed ionic bonds with Ca^2+^ [40], whereas flavonoids were adsorbed onto hydrophobic microdomains of SA via *π*–*π* stacking [41]. This surface layer serves as an additional physical barrier that impedes gastric acid and enzyme diffusion, while also reducing wall porosity to minimize probiotic leakage during storage and transit.

A comparison of Figure 4a,d revealed that microcapsules without PE exhibited pronounced surface depressions and cracks due to the low gel strength of pure SA, which was insufficient to withstand mechanical stress during freeze‐drying [42]. Such structural defects compromise barrier integrity, increasing permeability to gastric acid and leading to premature probiotic release and reduced viability. In contrast, PE‐FUA033 microcapsules maintained a smooth and continuous surface (Figure 4d,f), indicating that PE reinforced the gel network through additional cross‐linking interactions—both ionic coordination with Ca^2+^ and hydrogen bonding with SA chains—thereby increasing mechanical strength and structural resilience. Furthermore, as shown in Figure 4e, probiotics were uniformly dispersed within the polymer matrix in PE‐containing microcapsules, whereas controls exhibited uneven distribution and localized cell aggregation (Figure 4b). This uniform dispersion ensures that each bacterial cell is adequately protected by the surrounding wall material, maximizing encapsulation efficiency and providing consistent protection. The improved dispersion is likely mediated by the surface‐active properties of polyphenols, which reduce interfacial tension and prevent cell aggregation during cross‐linking. Additionally, bioactive components in PE (e.g., ellagic acid and gallic acid) protect probiotics by decreasing osmotic shock during cross‐linking and scavenging hydroxyl radicals, thereby reducing oxidative stress‐induced cell damage [43].

The incorporation of PE into the alginate‐Ca^2+^ matrix confers multiple structural and functional benefits. These combined effects directly translate into the improved storage stability and gastrointestinal survival observed in subsequent experiments, confirming that PE acts as an active structural enhancer that optimizes the protective performance of the microcapsule system.

#### 3.3.2. FTIR Analysis

**Figure 5:**
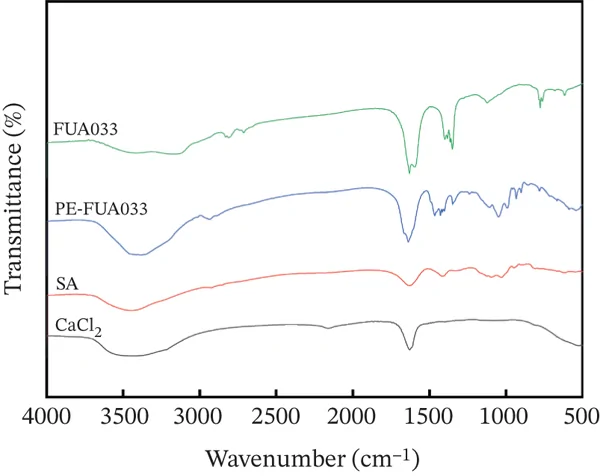
FTIR spectra of two types of microcapsules and raw materials.

Figure 5 shows the FTIR spectra of freeze‐dried SA, CaCl_2_, and microcapsules. In the SA spectrum, the peak at 3448 cm^−1^ is assigned to the O–H stretching vibration; the peaks at 1630 and 1414 cm^−1^ correspond to the asymmetric and symmetric stretching vibrations of the carboxylate group (–COO^−^), respectively [44]. The peak at 1030 cm^−1^ arises from the C–C and C–O skeletal vibrations. In the CaCl_2_ spectrum, the peak at 3433 cm^−1^ is the O–H stretching vibration, and the peak at 1630 cm^−1^ is the H–O–H bending vibration. In the spectrum of FUA033 microcapsules, the peak at 1632 cm^−1^ is the asymmetric stretching of –COO^−^, the peak at 1597 cm^−1^ is the C=O stretching, the peak at 1400 cm^−1^ is the symmetric stretching of –COO^−^, the peak at 1383 cm^−1^ is the C–H bending and C–N stretching vibrations, and the peak at 777 cm^−1^ is the C–O–C skeletal vibration of the sugar ring [31].

After cross‐linking of SA with Ca^2+^, characteristic shifts were observed in the spectrum of FUA033 microcapsules compared with SA: the peaks of –COO^−^ at 1630 and 1414 cm^−1^ shifted to 1632 and 1400 cm^−1^, and the sugar‐ring C–O–C vibration shifted from 793 to 777 cm^−1^, confirming the formation of an “egg‐box” cross‐linked structure [45], which originates from the coordination of Ca^2+^ with carboxyl and hydroxyl groups of guluronic acid [46]. The peak at 1639 cm^−1^ for PE‐FUA033 microcapsules showed a blue shift of 7 cm^−1^ toward higher wavenumbers compared with FUA033 microcapsules, indicating competitive coordination between polyphenolic hydroxyl groups in PE and Ca^2+^ [47]. The peak at 1466 cm^−1^ is attributed to the C=C skeletal vibration of ring B in pomegranate polyphenols, with a match > 95% to the spectrum of ellagic acid standard [48], indicating successful encapsulation of PE in the microcapsules. Peaks at 1109, 1049, and 995 cm^−1^ indicate significantly enhanced C–O–C glycosidic bond vibrations, reflecting the formation of a hydrogen‐bond network between polyphenolic hydroxyl groups in PE and sugar units of SA, which can improve the thermal stability of microcapsules.

#### 3.3.3. XRD Analysis

**Figure 6:**
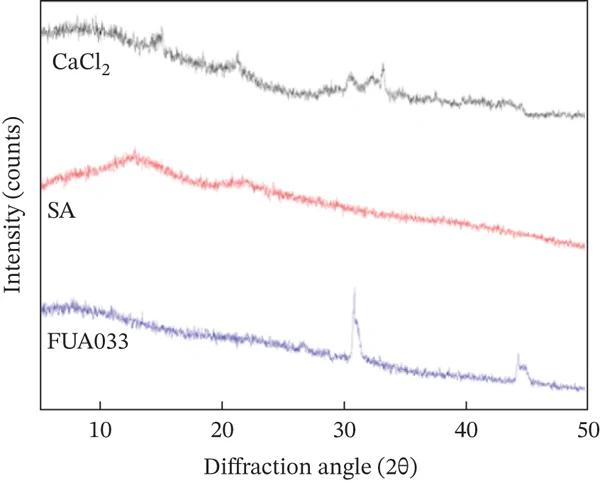
XRD patterns of microcapsules and raw materials.

The crystalline structures of raw materials and microcapsules were analyzed by XRD (Figure 6). SA exhibited typical amorphous peaks at 12.90° and 20.86°, characteristic of Type I hydrated alginate, whereas CaCl_2_ showed sharp diffraction peaks at 15.12°, 21.32°, and 33.2°, corresponding to the crystalline structure of CaCl_2_·2H_2_O. Upon microencapsulation, the FUA033 microcapsules displayed new sharp diffraction peaks at 30.86° and 44.28°, attributed to the formation of an ordered “egg‐box” cross‐linked structure resulting from Ca^2+^ coordination with guluronic acid units [30]. This crystalline ordering is functionally significant, as it increases the structural rigidity of the hydrogel matrix, thereby enhancing its resistance to mechanical disruption and enzymatic degradation during gastrointestinal transit.

Notably, compared with the raw CaCl_2_ peak at 15.12°, the corresponding peak in the FUA033 microcapsules shifted negatively to 13.48° (*Δ*2*θ* = −1.64°). This shift reflects lattice expansion caused by two competing mechanisms: the phenolic hydroxyl groups of polyphenols in PE compete with SA carboxyl groups for Ca^2+^ coordination, increasing interplanar spacing; and internal stress within the SA‐Ca^2+^–PE ternary network exerts a tensile effect on the crystal domains. The resulting diffraction pattern differed substantially from both raw materials, confirming that PE was successfully embedded within the three‐dimensional SA matrix and induced the formation of a novel organic–inorganic composite phase. This structural reorganization—neither purely amorphous like SA nor simply crystalline like CaCl_2_·2H2O—demonstrates that PE actively participates in network assembly rather than serving as a passive filler.

The sharp, narrow diffraction peaks of the PE‐FUA033 microcapsules indicate significantly enhanced crystallinity compared with the controls [49]. Higher crystallinity is directly associated with reduced water permeability and increased barrier function, as the ordered molecular packing limits the diffusion of small molecules such as gastric acid and bile salts. This rigid, well‐organized skeleton structure thus provides superior physical protection for encapsulated probiotics. Collectively, the XRD results confirm that PE is not merely encapsulated but actively reconstructs the crystal network through competitive coordination with Ca^2+^, ultimately forming a stable, high‐crystallinity composite phase that enhances the protective performance of the microcapsule system.

### 3.4. Storage Stability of Microcapsules

**Figure 7:**
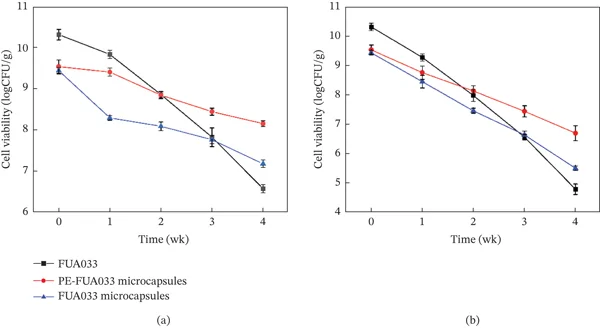
Storage stability of microcapsules and free cells at (a) 4°C and (b) 25°C.

The storage stability of probiotic formulations is critical for maintaining their efficacy throughout commercial distribution and consumer use. We evaluated the viability of free FUA033 cells, FUA033 microcapsules (without PE), and PE‐FUA033 microcapsules during 4 weeks of storage at refrigerated 4 ± 1°C and ambient 25 ± 1°C temperatures (Figure 7).

After 4 weeks at 4°C, free cells declined sharply from 10.31 to 6.57 log CFU/g. Microencapsulation without PE significantly improved retention (7.18 log CFU/g), while PE‐FUA033 microcapsules maintained the highest viability (8.15 log CFU/g). This protective effect was even more pronounced at the more stressful ambient temperature: free cells decreased to 5.51 log CFU/g, whereas PE‐FUA033 microcapsules retained 6.80 log CFU/g. Notably, only PE‐FUA033 microcapsules maintained viable counts above the therapeutic threshold of 10^6^ CFU/g after 4 weeks at 25°C, highlighting their practical applicability for nonrefrigerated products.

The enhanced stability conferred by PE arises from multiple synergistic mechanisms. First, the polyphenolic compounds in PE (e.g., ellagic acid and gallic acid) act as potent antioxidants, scavenging free radicals generated during storage and thereby reducing oxidative damage to bacterial cell membranes and proteins [43]. Second, these bioactive components may function as prebiotics, providing metabolic substrates that sustain bacterial viability under nutrient‐limited conditions [50]. Third, as confirmed by FTIR and XRD analyses, PE incorporation creates a denser, more ordered alginate network with reduced oxygen permeability—directly limiting the diffusion of oxidative stressors into the microcapsule interior [51]. The greater relative protection observed at 25°C is mechanistically significant: elevated temperatures accelerate both oxidative reactions and bacterial metabolic stress, making the antioxidant capacity of PE critically important under ambient storage conditions.

These findings demonstrate that the PE‐FUA033 microcapsule system effectively maintains probiotic viability across different storage temperatures by combining the physical barrier function of the alginate‐Ca^2+^ matrix with the biochemical protection afforded by pomegranate polyphenols. The retention of > 10^6^ CFU/g after 4 weeks at ambient temperature underscores the commercial potential of this formulation for products requiring nonrefrigerated distribution. By integrating physical and chemical protection strategies, this approach offers a robust platform for developing highly stable probiotic preparations with extended shelf life.

### 3.5. Gastrointestinal Digestion of Microcapsules

**Figure 8:**
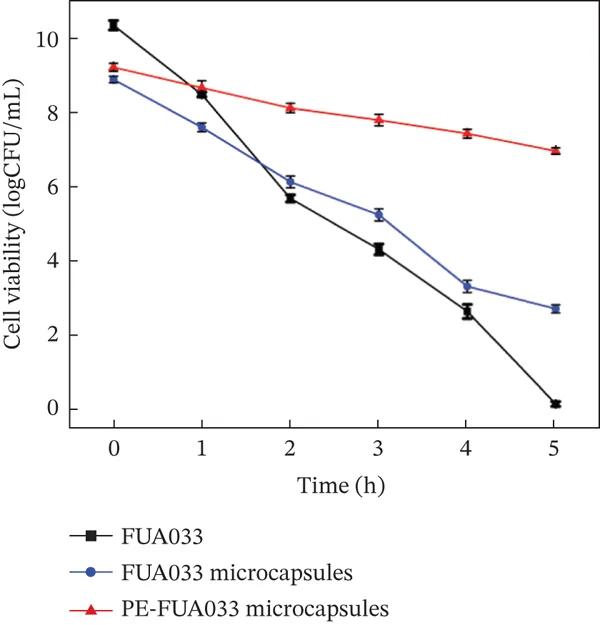
Cell viability of microcapsules and free cells after simulating the gastrointestinal digestion process.

The efficacy of probiotic microcapsules ultimately depends on their ability to deliver viable cells to the intestine. Using an in vitro simulated gastrointestinal digestion model, we compared the survival of free FUA033 cells, FUA033 microcapsules (without PE), and PE‐FUA033 microcapsules through sequential gastric and intestinal phases (Figure 8).

After 2 h of simulated gastric digestion, free cells were almost completely eliminated. FUA033 microcapsules retained 6.11 log CFU/g, demonstrating the protective effect of alginate encapsulation. Remarkably, PE‐FUA033 microcapsules maintained 8.09 log CFU/g. Following subsequent intestinal digestion, the viable count of FUA033 microcapsules plummeted to 2.71 log CFU/g, falling below the therapeutic threshold of 10^6^ CFU/g. In contrast, PE‐FUA033 microcapsules retained 6.94 log CFU/g, remaining well above this critical threshold throughout the entire digestion process. These data demonstrate that PE confers not only immediate gastric protection but also sustained protective effects during intestinal transit.

This enhanced protection arises from multiple complementary mechanisms that operate at different levels. First, structural reinforcement: As evidenced by SEM, FTIR, and XRD analyses, PE incorporation creates a denser, more ordered alginate network with reduced porosity. Polyphenols participate in additional cross‐linking with Ca^2+^ and form hydrogen bonds with alginate chains, increasing network density and mechanical strength [51]. This reinforced matrix more effectively impedes the diffusion of gastric acid and bile salts into the microcapsule interior. Second, uniform cell distribution: The surface‐active properties of polyphenols promote uniform dispersion of probiotics within the matrix [52], ensuring that each cell is surrounded by an adequate protective layer—unlike the uneven distribution and localized aggregation observed in PE‐free controls (Figure 4b). Third, biochemical protection: In addition to enhancing the stability of microcapsules, the polyphenolic compounds in PE act as intracellular antioxidants, scavenging reactive oxygen species generated during digestive stress and preserving metabolic activity. Additionally, as potential prebiotics, these compounds may provide metabolic substrates that sustain bacterial viability under the nutrient‐limited conditions of the gastrointestinal tract [53].

The synergistic interplay between physical barrier function and biochemical protection explains the exceptional performance of PE‐FUA033 microcapsules. The physical matrix provides the first line of defense against gastric acid, whereas the antioxidant and prebiotic activities of PE mitigate cellular damage at the molecular level—particularly important during the prolonged intestinal phase where oxidative stress predominates. The retention of > 10^6^ CFU/g through complete simulated digestion confirms that this formulation meets the minimum requirement for probiotic efficacy, positioning it as a promising candidate for functional food applications requiring targeted intestinal delivery.

## 4. Conclusion

In this study, a sodium alginate–calcium chloride hydrogel microcapsule system was constructed for coloading pomegranate active extract with the probiotic FUA033. Through systematic optimization of key formulation parameters—including sodium alginate concentration, calcium chloride concentration, PE concentration, and curing time—the encapsulation efficiency was significantly enhanced, reaching 90.23% under optimal conditions. Comprehensive physicochemical characterization (SEM, FTIR, and XRD) confirmed that the incorporation of PE not only preserved the structural integrity of the alginate‐Ca^2+^ matrix but also actively reinforced it. The formation of a denser surface layer, additional cross‐linking interactions, and increased crystallinity collectively contributed to enhanced barrier function and mechanical stability. These structural improvements directly translated into superior functional performance: PE‐FUA033 microcapsules maintained significantly higher probiotic viability during 4 weeks of storage at both refrigerated and ambient temperatures, and demonstrated markedly improved survival through simulated gastrointestinal digestion. The resulting codelivery system not only addresses the critical challenge of maintaining probiotic viability during gastrointestinal transit but also provides a strategy for valorizing pomegranate processing by‐products. This study establishes a scientific foundation for the development of next‐generation synbiotic formulations that leverage the synergistic interactions between probiotics and plant‐derived polyphenols. Future work may explore the in vivo efficacy of this system and its potential for delivering other combinations of bioactive compounds and probiotic strains.

## Author Contributions

Yanzi Ge: writing—original draft, writing—review and editing, formal analysis, conducted the experiments, analyzed the data, and drafted the manuscript. Jiayi Hu: formal analysis, investigation, and performed the extraction and microencapsulation experiments. Shu Liu: resources, supervision, provided critical guidance on experimental design, particularly optimization of microencapsulation parameters and structural characterization, and contributed to interpretation of results. Guang Yang: conceptualization, project administration, resources, contributed to conceptualization of the microcapsule codelivery system, and provided supervision. Qingyun Hua: formal analysis, software, performed statistical analysis, and prepared figures. Qiran Gu: methodology, investigation, and assisted in microcapsule preparation and characterization. Tianyi Mao: data curation, validation, validated experimental data and curated datasets. Xiaoxu Xue: visualization, investigation, and assisted in SEM and FTIR analyses. Bo Yu: methodology, supervision, contributed to design of in vitro simulated digestion experiments and analysis of gastrointestinal protection data, and ensured methodological rigor and reproducibility. Yaowei Fang: resources, writing—review and editing, conceived the overall research idea, designed the experimental framework, supervised the entire project, acquired the funding supporting this study, and contributed to interpretation of data and final manuscript approval.

## Funding

This study was supported by National Natural Science Foundation of Jiangsu Province (BK20210923); the Priority Academic Program Development of Jiangsu Higher Education Institutions (SZ202511641638002); and College Students′ Innovation and Entrepreneurship Training Program of Jiangsu Province (SY202511641638003).

## Conflicts of Interest

The authors declare no conflicts of interest.

## Data Availability

Data will be made available on request.
